# Evaluation of Smoking Status Identification Using Electronic Health Records and Open-Text Information in a Large Mental Health Case Register

**DOI:** 10.1371/journal.pone.0074262

**Published:** 2013-09-12

**Authors:** Chia-Yi Wu, Chin-Kuo Chang, Debbie Robson, Richard Jackson, Shaw-Ji Chen, Richard D. Hayes, Robert Stewart

**Affiliations:** 1 Department of Nursing, College of Medicine, National Taiwan University, Taipei, Taiwan; 2 Department of Health Service & Population Research, Institute of Psychiatry, King’s College London, London, United Kingdom; 3 Mackay Memorial Hospital Taitung Branch, Taitung, Taiwan; 4 School of Medicine, Buddhist Tzu Chi University, Hualien, Taiwan; The University of Texas M. D. Anderson Cancer Center, United States of America

## Abstract

**Background:**

High smoking prevalence is a major public health concern for people with mental disorders. Improved monitoring could be facilitated through electronic health record (EHR) databases. We evaluated whether EHR information held in structured fields might be usefully supplemented by open-text information. The prevalence and correlates of EHR-derived current smoking in people with severe mental illness were also investigated.

**Methods:**

All cases had been referred to a secondary mental health service between 2008-2011 and received a diagnosis of schizophreniform or bipolar disorder. The study focused on those aged over 15 years who had received active care from the mental health service for at least a year (N=1,555). The ‘CRIS-IE-Smoking’ application used General Architecture for Text Engineering (GATE) natural language processing software to extract smoking status information from open-text fields. A combination of CRIS-IE-Smoking with data from structured fields was evaluated for coverage and the prevalence and demographic correlates of current smoking were analysed.

**Results:**

Proportions of patients with recorded smoking status increased from 11.6% to 64.0% through supplementing structured fields with CRIS-IE-Smoking data. The prevalence of current smoking was 59.6% in these 995 cases for whom this information was available. After adjustment, younger age (below 65 years), male sex, and non-cohabiting status were associated with current smoking status.

**Conclusions:**

A natural language processing application substantially improved routine EHR data on smoking status above structured fields alone and could thus be helpful in improving monitoring of this lifestyle behaviour. However, limited information on smoking status remained a challenge.

## Introduction

Smoking behaviour is an important potential contributor to morbidity and premature mortality in people with severe mental illness (SMI) [1]. Studies of smoking characteristics and prevalence in people with SMI have mostly focused on people with diagnoses of schizophrenia and bipolar disorder [2]. UK prevalences of smoking in people with SMI have ranged between 49-65% [3-5]. In a meta-analysis of 42 studies published between 1983 and 2005, the pooled smoking prevalence was 62% in 7,593 patients with schizophrenia [6]. Smoking was more prevalent in those recruited from psychiatric inpatient settings (68%) compared to those in community settings (57%). de Leon, and Diaz (2005) calculated a pooled odds ratio for smoking of 5.3 (95% CI: 4.9 to 5.7) comparing people with schizophrenia to the general population; whereas the odds ratio was 1.9 (95% CI: 1.7 to 2.1) for bipolar disorder [6]. People with SMI were more likely to be heavier smokers and more nicotine-dependent than smokers in the general population [7]. Heavy smoking in patients with schizophrenia has been reported to be associated with more positive symptoms, increased substance misuse, more frequent psychiatric hospitalization and a higher suicide risk [8].

The socioeconomic determinants of smoking behaviour have been extensively studied in the general population. Smoking prevalence in the UK is highest in the 20-24 age group (28%) and lowest in people over 60 (13%) [9]. Recent studies find that men and women now have similar smoking prevalences in the general population in the UK (21% and 20%), and people who are married have much lower smoking prevalences compared to those who are single or divorced [9]. Higher prevalences of smoking are found in people with lower levels of education [10], and smoking is strongly associated with unemployment [11]. Demographic and socioeconomic determinants of smoking may differ between SMI and the general population, although data on this have been limited. One study found that gender differences in smoking behaviour were more marked in SMI than in the general population in that males with schizophrenia were 2.5 times more likely to smoke than women [6,12]. Rather than comparing smoking prevalence in patients with schizophrenia to the general population, some researchers have argued that patients with other disorders underlying SMI would be a more appropriate comparison group [6,13], because of the problem of confounding.

The first step in treating tobacco dependence is to identify tobacco users. Electronic health records (EHRs) have the potential to provide informative and longitudinal data on smoking status in people receiving health care. This is important both for informing and improving the assessment of smoking behaviour, and for developing service-level strategies for smoking cessation support. Routine data can cost-effectively facilitate the evaluation of smoking cessation interventions and track trends over time. Smoking status records in secondary care EHRs provide potential supplementary data to those in primary care and such data have been used for monitoring a range of health conditions and behaviours including acute respiratory infections [14] and colonoscopy quality [15], as well as smoking [16]. However, the utility of these data may be challenged by issues of documentation quality and generalisability [17]. Structured fields in EHRs can be used as a source of smoking related information, but there are limitations in the applicability of check boxes in routine clinical practice, particularly for situations such as smoking behaviour where repeated measures may be required for effective surveillance rather than a single collection of information at service entry. Combining structured data with information derived from open-text fields in the EHRs has been found to improve sensitivity and precision in some clinical scenarios [14], but has received relatively little evaluation in mental health care settings for smoking or any other exposure.

The primary aim of the study was to investigate smoking prevalence and factors influencing this in people receiving mental healthcare, evaluating the derivation of this information from both structured and open-text records fields of a large EHR-sourced database: the South London and Maudsley (SLaM) Case Register.

## Materials and Methods

The Case Register study received ethical approval as an anonymised data resource for secondary analyses by Oxfordshire REC C in 2008 (reference number 08/H0606/71). The SLaM Case Register was established in 2006 and has been used for a range of research projects focusing on SMI and its consequences [18-22] with a robust de-identification program [22]. SLaM is the largest unit provider of secondary mental healthcare in Europe, covering a socially diverse geographic catchment of 1.2 million residents in southeast London. Fully electronic health records were implemented across all SLaM services and the Clinical Record Interactive Search (CRIS) system was built up in 2008 allowing researcher to access to full but de-identified records on an overnight basis. Currently, records of over 200,000 mental health service users are available for research. Individual consent was not obtained for this study because data had been effectively anonymised by CRIS prior to researcher access, in compliance with European data protection law. Recent developments of the CRIS data resource have included the application of natural language processing applications to derive structured data from the extensive volumes of open-text contained in a standard mental health case record, several of which are currently submitted for publication.

The study sample derived from the SLaM Case Register comprised a cohort of all patients who had received a diagnosis of SMI (comprising schizophrenia (ICD-10 code: F20-24), schizoaffective disorder (F25), and bipolar disorder (F30-31)), who had been referred to SLaM between 1^st^ January 2008 and 31^st^ December 2011, and who were aged over 15 years old at referral. It was assumed that there would be higher likelihood of data on smoking in cases who had received more mental health service inputs over a longer period of time, and a further sub-sample were therefore analysed who had been receiving active care and/or follow-up from SLaM for at least twelve months from referral.

**Figure 1:**
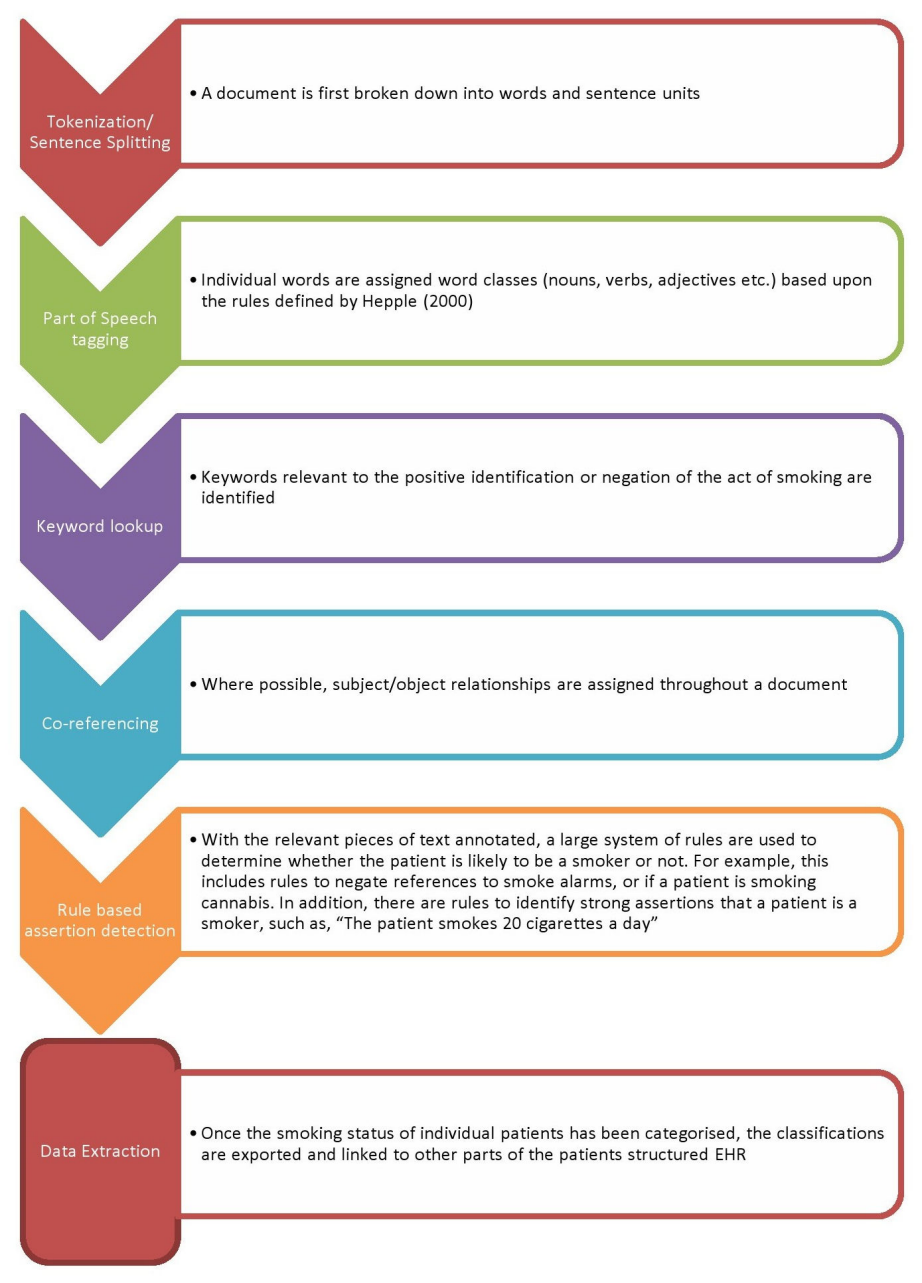
Free-text data extraction algorithm.

Open-text fields in the Case Register including written assessments, progress notes and correspondence were interrogated using natural language processing software, General Architecture for Text Engineering (GATE). In collaboration with the University of Sheffield and Ontotext, we developed the CRIS-IE-Smoking application on the GATE platform to determine the smoking status of individuals. The application extracts information from the abovementioned open-text fields, classifying patients as either ‘currently smoking’, ‘past smoker’ or ‘has never smoked’, with smoking of substances other than tobacco (e.g. marijuana / cannabis and cocaine) specifically excluded. CRIS-IE-Smoking employs a shallow parsing, rule-based approach, based on the absence/presence of certain keywords within the electronic record. The rules, summarised in Figure 1, were developed using an iterative process of manual ‘gold standard’ annotation of free text documents, followed by comparison with the results generated by the application at each development stage, with analysis of this comparison feeding further development of the rules developed by Hepple [23]. This process was repeated until a precision (positive predictive value) of 93% for automatic annotations from 100 random documents was achieved. The annotation-level recall (sensitivity) at this level was 58%. The application was developed to maximise annotation-level precision because of the assumed repetition of information across documents for each patient (i.e. improving recall). The CRIS-IE-Smoking is available as open source software on https://sourceforge.net/projects/crisiesmoking/, and requires a copy of GATE, available freely at http://gate.ac.uk/. We are unable to place test data in the public domain because these comprise patient information, but these have been archived and researchers may apply for approval to access these or other CRIS data. More information is available at http://brc.slam.nhs.uk/about/core-facilities/cris.

Proportions of cases for whom smoking data were available and the prevalence of current smoking in that sample were calculated for the total sample and for the cases who had been receiving care from the service provider for at least twelve months, smoking status being ascertained initially just from structured fields, followed by estimates using post-processed open-text data. The first record of smoking status after referral was the focus for all analyses. Prevalences were further investigated for the individual years in which cases had been referred and then by sociodemographic characteristics including age, gender, marital status, deprivation scores, and the included components of ICD-10 diagnoses, followed by logistic regression analyses to assess factors associated with current smoking status in the period of study.

## Results

The analysed sample comprised 5,588 cases overall, 1,555 of whom received care for at least 12 months under SLaM. As described in Table 1, the use of structured data alone provided information on smoking status for very low proportions of cases, whereas this was increased substantially (over five-fold) when combined with data from processed text. Highest coverage of cases (around 64%) was found in those who had received active mental health care for at least 12 months and where information was drawn from both open-text and structured fields. Further analysis by year of referral (omitting 2011 referrals because of being unable to specify at least 12 months of care receipt), these proportions appeared stable (Table 2). Missing smoking annotations did not vary by marital status, but were most common in 35-64 year olds, in women, in areas with lower deprivation scores, as well as in people with bipolar disorder (Table 3).

**Table 1:** Presence of information on smoking status in the electronic health record sourced database according to information source.

Information source for smoking status	Number (%) with any information on smoking status	
	Total sample (n=5,588)	Cases receiving active mental health care for at least 12 months (n=1,555)
Structured field only	547 (9.8)	180 (11.6)
Structured field plus post-processed open-text information	2907 (52.0)	995 (64.0)

**Table 2:** Presence of information on smoking over study years (2008-2010).

Year of referral	Total sample (n=5,588)			Sample receiving at least 12 months mental healthcare (n=1,555)		
	Number	% with smoking annotations		Number	% with smoking annotations	
		Using structured field only	Using all information		Using structured field only	Using all information
2008	1,493	10.1	54.9	504	10.7	64.1
2009	1,483	8.7	51.9	499	12.2	64.9
2010	1,396	9.4	53.6	512	11.5	62.9
p-value*		0.49	0.45		0.69	0.69

Cochran-Armitage chi-squared test for linear trend on one degree of freedom

**Table 3:** Factors associated with missing smoking information patients receiving active mental healthcare for at least twelve months (n=1,555).

		Number in category	Number (%) with missing smoking information	p-value
Age group	15-24	364	113 (31.0)	<0.001* 0.18**
	25-34	429	156 (36.4)	
	35-44	317	140 (44.2)	
	45-54	179	76 (42.5)	
	55-64	92	42 (45.7)	
	65-74	85	20 (23.5)	
	75+	89	13 (14.6)	
Gender	Male	802	247 (30.8)	<0.001*
	Female	753	313 (41.6)	
Marital status	Single	1019	345 (33.9)	0.19*
	Cohabiting	270	106 (39.3)	
	Separated/widowed	214	69 (32.2)	
Deprivation score in tertiles	Least deprived	473	199 (42.1)	0.004* 0.002**
	Middle	502	171 (34.1)	
	Most deprived	465	150 (32.3)	
Primary diagnosis	Schizophrenia	669	213 (31.8)	<0.001*
	Schizoaffective	62	20 (32.3)	
	Bipolar disorder	384	189 (49.2)	
	Other	440	138 (31.4)	

^*^Pearson chi-squared test for heterogeneity

^**^Cochran-Armitage chi-squared test for linear trend on one degree of freedom

Factors associated with current smoking status were investigated in the 995 cases for whom this information was available and who had received active mental healthcare for at least 12 months, results of which are summarised in Table 4. Of this sample, current smoking was recorded in 593 (59.6%), was most common in working age adults with a substantial fall in prevalence in post-retirement age ranges, and was more common in men compared to women, in people living in more deprived areas, and in single compared to cohabiting or separated/widowed cases. It was also more prevalent in cases with schizophrenia or schizoaffective disorder than in those with bipolar disorder. In logistic regression models with all covariates simultaneously entered, most associations were attenuated although those with age, gender, ‘other’ diagnosis, and marital status remained significant (Table 5).

**Table 4:** Factors associated with current smoking status in cases receiving active mental healthcare for at least 12 months with smoking status recorded (n=995).

		Number in category	Number (%) current smokers	p-value
Age group	15-24	251	172 (68.5)	<0.001* <0.001**
	25-34	273	170 (62.3)	
	35-44	177	109 (61.6)	
	45-54	103	69 (67.0)	
	55-64	50	33 (66.0)	
	65-74	65	18 (27.7)	
	75+	76	22 (29.0)	
Gender	Male	555	370 (66.7)	<0.001*
	Female	440	223 (50.7)	
Marital status	Single	674	446 (66.2)	<0.001*
	Cohabiting	164	72 (43.9)	
	Separated/widowed	145	68 (46.9)	
Area-level deprivation score in tertiles	Least deprived	274	147 (53.7)	0.023* 0.006**
	Middle	331	194 (58.6)	
	Most deprived	315	204 (64.8)	
Primary diagnosis	Schizophrenia	456	297 (65.1)	0.003*
	Schizoaffective	42	28 (66.7)	
	Bipolar	195	110 (56.4)	
	Others	302	158 (52.3)	

^*^Pearson chi-squared test for heterogeneity

^**^Cochran-Armitage chi-squared test for linear trend on one degree of freedom

**Table 5:** Logistic regression analysis of factors associated with current smoking status in cases receiving mental healthcare for at least 12 months after referral with smoking status recorded (n=995).

Variables		Unadjusted	Mutually adjusted
Age 65 or above		0.22 (0.15-0.32)	0.23 (0.14-0.37)
Female gender		0.51 (0.40-0.66)	0.62 (0.46-0.83)
Marital status	Single	Ref	Ref
	Cohabiting	0.40 (0.28-0.57)	0.53 (0.36-0.79)
	Separated/widowed	0.45 (0.31-0.65)	1.00 (0.62-1.62)
Deprivation score (per tertile increase)		1.26 (1.07-1.49)	1.26 (1.05-1.51)
Psychiatric diagnosis	Schizophrenia	Ref	Ref
	Schizoaffective disorder	1.07 (0.55-2.09)	1.66 (0.79-3.51)
	Bipolar disorder	0.69 (0.49-0.98)	0.77 (0.52-1.13)
	Others	0.59 (0.44-0.79)	0.58 (0.42-0.81)

## Discussion

The results showed a greater than five-fold higher identification rate of smoking status in people with severe mental illness through supplementing routine structured fields in a large mental health electronic record system with information from processed open-text from the same record. Smoking status could thus be routinely identified at least on one occasion in 52% of all referrals and 64% of referrals with a longer (12+ months) care pathway. Analysing the latter group, prevalence of current smoking was close to 60% and was more common in pre-retirement age groups, in men, in single people and in those living in less affluent areas (i.e. higher levels of deprivation). Moreover, the prevalence varied significantly by diagnostic group and was most common in schizophrenia and schizoaffective disorder. However, many of the groups with fewer smokers (women, people with bipolar disorder, people living in more affluent areas) were also those in whom data on smoking status were more likely to be missing.

Prevalence of smoking in our sample was similar to estimates from inpatients with schizophrenia in Scotland [3], where 58% were current smokers (compared to 27% in the general population). It was higher than the 49% prevalence found in 89 patients with new onset psychosis in South London [5], but was lower than the 65% prevalence of current smoking among people with schizophrenia reported by McCreadie et al [4] (compared to 40% in their local general population). They are also similar to the 62% smoking prevalence in a meta-analysis of 42 studies of people with schizophrenia across 20 countries [6]. Patients with schizophrenia and schizoaffective disorder had higher smoking prevalences than those with a bipolar disorder, which is similar to findings from other studies [6,13], and also in line with other studies, the prevalence in our sample was over three times higher than general population estimates which are currently 17% for London residents [9]. Although it has been argued that high prevalences of smoking in people with schizophrenia are cited more often than studies with lower estimates creating citation bias [24], our study lends support to mounting evidence of concerning high smoking prevalence among people with SMI.

The associations of current smoking with younger age, male gender, and lower socio-economic status are also consistent with other research [25,26]. Men in our sample were more likely to be smokers compared to women, although the differences were not as marked as in other studies [6,12]. These findings indicate that smoking in this disorder group is more prevalent among the young and middle-aged cases, particularly men and those who live in less affluent areas or who are single. However, caution should be exercised in the interpretation of the findings because missing data on smoking status was not random, but was associated with gender, area-level socioeconomic status and diagnosis. The fact that it was not differential by age group or marital status renders selection bias less likely to account for all primary associations; however, an influence of differential ascertainment cannot be ruled out absolutely.

With growing awareness of the importance of poor physical health as a cause of premature mortality in SMI, there is an increasing need for the most salient risk factors for poor health to be monitored and interventions developed and targeted for risk reduction strategies. Tobacco use is the largest single preventable cause of disease, disability and death, and methods for improving the assessment and identification of smokers who use mental health services is an essential first step in developing a local strategy to encourage smokers to quit. While ascertainment of smoking status is relatively straightforward at an individual level, the adequate acquisition of data over large clinical populations is more challenging because this relies on routine data being recorded and made available for analysis. However, availability of these data remain important both for cross-sectional/retrospective analyses (e.g. investigating sub-groups with particularly high smoking prevalence for targeting interventions) and for prospective research (e.g. monitoring of changes in prevalence following service-level interventions). A number of ways have been found to improve the identification and recording of tobacco use. Recording smoking status at the same time as monitoring vital signs (blood pressure, temperature, and pulse rate) was found to have a modest effect on identification [27]. Financial incentives have proved effective in increasing the rate of documentation and stop smoking advice. Since the introduction in 2004 of the Quality and Outcomes Framework (QOF), a voluntary pay-for-performance general practice contract, GPs in Britain are required to record patients’ smoking status and treatment offered, at least every 15 months for people with long term conditions (including schizophrenia and bipolar disorder). The recording of smoking status in patient’s medical records and related QOF targets increased considerably immediately following the introduction of the QOF, although rates of compliance have plateaued in recent years [16]. An alternative approach – i.e. to improve structure of EHRs, through routine processing of open-text has the potential advantage of not requiring additional time devoted to record keeping. One way of ascertaining smoking status by routinely recording smoking status in the open-text records would be to apply the natural language processing approach in the EHRs, which may reduce the risk of under-estimating patient smoking status resulted from missing data in the tick-box A key disadvantage of the above approach is in a loss of accuracy since no natural language processing application is likely to achieve perfect ascertainment. However, this is to some extent ameliorated both through the large case numbers which can be assembled in routine clinical practice through the analysis of electronic health records, as well as the advantage for an exposure such as current smoking of repeated occurrences of relevant text throughout the record (e.g. sub-optimal sensitivity at the level of an individual annotation or text field is compensated for where there are multiple records). It thus has advantages for monitoring trends over large samples but is probably less suited for purposes focused on the individual.

As described, we found that the overall ascertainment of smoking status increased substantially when routine structured fields within the electronic health record were supplemented by automated extractions from open-text fields. This supports findings in other areas that combining multiple data sources achieves better performance on disease or risk status identification [14,28,29]. However, our 59% prevalence finding combining open-text and structured data is lower than that from a data analysis strategy involving only open-text [30]. The natural language processing approach used here is likely to be enhanced as advances in machine learning are increasingly applied in health service data [31], and in the era of personalized medicine such an approach towards disease risk identification may also enhance healthcare cost-effectiveness by identifying high risk groups for early interventions. For the development of smoking cessation services within mental healthcare, underlying EHR data integration provides a valuable opportunity for facilitating case identification. Improving the recording of smoking status has the potential to increase clinician interventions, however systems changes beyond smoker identification strategies are likely to be needed to increase the rate of cessation advice and intervention [32].

Strengths of this study include the large sample and routine data drawn from services which had not received any prior intervention or directive to promote smoking status ascertainment, so that findings are likely to reflect practice at the time of the analysis. Considering generalisability, although this study was restricted to a single organisation’s record data, between-service variability within SLaM for smoking status ascertainment is likely to reflect levels of variability elsewhere, in the UK at least. Fully electronic health records are now routine in UK mental health service providers, just as they are in primary care. Although records systems vary, the application of natural language processing in open-text is unlikely to need substantial modification to process records from different services and on different platforms. The catchment area covers both inner urban and suburban environments but does not contain any rural population. The key limitation of missing data on smoking status has been considered and should be borne in mind when interpreting findings. The level of missing rate of smoking was higher than the one-quarter missing rate of that previously described in a study of primary care electronic medical records [33]. As well as pursuing potential avenues for improving the natural language processing application, an important implication is that clinical services should pay more attention to smoking status, but that this might involve relatively light-touch changes to the way in which correspondence and case notes are structured on the electronic record, rather than the imposition of a compulsory check-box. Another limitation was that analyses of factors associated with current smoking status were restricted to cases who were receiving input from the service provider for at least twelve months, restricting generalizability to those with shorter care episodes. The focus was on current smokers within specific diagnostic groups and no attempt was made at this stage to ascertain smoking history, or indeed other illicit drugs such as cannabis whose consumption may be combined with tobacco.

## Conclusions

Our results demonstrate an achievable and substantial improvement in smoking identification in mental healthcare electronic health records with preliminary data on patterns and correlates of smoking behaviour in SMI as well as patterns of non-ascertainment. We believe that these findings support wider research and development in the field to improve surveillance of risk behaviours in this group known to be vulnerable to adverse health outcomes. 
